# Primary care clinician antibiotic prescribing decisions in consultations for children with RTIs: a qualitative interview study

**DOI:** 10.3399/bjgp16X683821

**Published:** 2016-02-08

**Authors:** Jeremy Horwood, Christie Cabral, Alastair D Hay, Jenny Ingram

**Affiliations:** Centre for Academic Primary Care, NIHR School of Primary Care Research, School of Social and Community Medicine.; Centre for Academic Primary Care, NIHR School of Primary Care Research, School of Social and Community Medicine.; Centre for Academic Primary Care, NIHR School of Primary Care Research, School of Social and Community Medicine.; Centre for Child and Adolescent Health, Schoolof Social and Community Medicine, University of Bristol, Bristol.

**Keywords:** antibiotics, childhood cough, diagnosis, qualitative research, respiratory tract infections

## Abstract

**Background:**

Respiratory tract infections (RTIs) are a major primary care challenge in children because they are common and costly, there is uncertainty regarding their diagnosis, prognosis, and management, and the overuse of antibiotics leads to illness medicalisation and bacterial resistance.

**Aim:**

To investigate healthcare professional (HCP) diagnostic and antibiotic prescribing decisions for children with RTIs.

**Design and setting:**

Semi-structured interviews conducted with 22 GPs and six nurses. HCPs were recruited from six general practices and one walk-in centre, serving a mix of deprived and affluent areas.

**Method:**

Interviews were audiorecorded, transcribed, imported into NVivo 9, and analysed thematically.

**Results:**

HCPs varied in the symptom and clinical examination findings used to identify children they thought might benefit from antibiotics. Their diagnostic reasoning and assessment of perceived clinical need for antibiotics used a dual process, combining an initial rapid assessment with subsequent detailed deductive reasoning. HCPs reported confidence diagnosing and managing most minor and severe RTIs. However, residual prognostic uncertainty, particularly for the intermediate illness severity group, frequently led to antibiotic prescribing to mitigate the perceived risk of subsequent illness deterioration. Some HCPs perceived a need for more paediatrics training to aid treatment decisions. The study also identified a number of non-clinical factors influencing prescribing.

**Conclusion:**

Prognostic uncertainty remains an important driver of HCPs’ antibiotic prescribing. Experience and training in recognising severe RTIs, together with more evidence to help HCPs identify the children at risk of future illness deterioration, may support HCPs’ identification of the children most and least likely to benefit from antibiotics.

## INTRODUCTION

Children aged <12 years have 4–11 acute respiratory tract infections (RTIs) per year^1^ and this infection is the commonest reason why parents consult primary care in the UK.^2^ Consultations for RTIs are more complex than most guidelines assume, requiring primary care doctors and nurses (from here on ‘healthcare professionals’, HCPs) to manage clinical uncertainty regarding diagnosis, prognosis, and treatment.^3^ Although antibiotics have only marginal beneficial effects on RTIs,^4^ they remain frequently prescribed, contributing to illness medicalisation^5^ and antibiotic resistance.^6^

Antibiotic prescribing rates in the UK declined during the late 1990s^7^ but then rates levelled off and began to increase again in the early 2010s.^7^,^8^ Previous qualitative research examining the influences of antibiotic prescribing for children with acute illness has focused on unnecessary prescribing, giving less attention to clinical factors.^9^ It has been reported that clinicians may prescribe ‘just in case’ when they were uncertain about the clinical or social outcomes of not prescribing and were more likely to prescribe if they perceived pressure from parents.^9^ Parents, however, are primarily seeking a medical evaluation and many have a no treatment preference.^9^–^11^ Clinicians can be mistaken in the perception of parent pressure^12^ and this can sometimes lead to unwanted and unnecessary antibiotic prescribing.^13^ Diagnostic complexity^3^ and prognostic uncertainty^14^ play a key role in antibiotic prescribing decisions for adults,^3^ and some prescribing practices are not well supported by the existing evidence base.^15^ However, there is a need to understand how HCPs decide to prescribe antibiotics for children with acute RTIs, taking into account both clinical and non-clinical influences. This study examined how HCPs make diagnostic and antibiotic prescribing decisions in consultations for children with RTIs.

## METHOD

Semi-structured interviews were conducted with HCPs (GPs and nurses who could prescribe or dispense antibiotics) recruited from six general practices and one walk-in-centre. Purposive sampling was used to capture maximum variation in experience. Practices serving a range of deprived and affluent areas, using the practice-level indices of multiple deprivation (IMD) scores,^16^ from both rural and urban areas, were purposively sampled to encompass different patient populations. From those practices, a researcher contacted HCPs about taking part in an interview. From those who agreed, a purposive sample (in relation to role, length of service, and paediatric experience) was recruited.

**How this fits in**

Previous research has focused on the influence of parental pressure on unnecessary antibiotic prescription. However, this study found that clinicians reported that parent pressure was rare and was outweighed by prognostic uncertainty and the non-clinical factors influencing antibiotic prescribing decisions in children. More detailed evidence about the prognosis of respiratory tract infections in children, especially those of intermediate illness severity, is required to support healthcare professionals to identify those children most and least likely to benefit from antibiotics.

Interviews were conducted by one researcher at the HCP’s workplace and lasted between 22 and 95 minutes. Interview topic guides explored HCPs’ experiences of consultations for children with RTIs, and the decision-making process in relation to diagnosis and disease management.

With written informed consent, interviews were audiorecorded, transcribed, and imported into NVivo 9 to aid data analysis. Data collection and analysis were conducted in parallel. Preliminary findings from early interviews were explored in later interviews. Interviewing continued until data saturation was reached and no new themes were arising from the data.^16^ Thematic analyses^17^ identified issues of particular salience for participants and across the dataset, using the constant comparison technique.^18^ The data were initially coded and a subset of interview transcripts were independently analysed, to contribute to the generation and refinement of codes to maximise rigour.^20^ A consensus about the final list of themes was reached through discussion among the qualitative research team.

## RESULTS

Twenty-two GPs and six nurses participated in interviews, with between 6 months to 35 years’ experience in primary care (Table 1). Analysis led to the development of the key emergent themes that concerned: clinical assessment and diagnostic process; uncertainty in diagnosis, prognosis, and prescribing; and non-clinical influences on antibiotic prescribing.

**Table 1.** Interview participants

	**GPs**	**Nurses**	**Total**
*N*	22	6	28

**IMD of practice population neighbourhood**			
1 (most deprived)	6	2	8
2	8	3	11
3	0	0	0
4	4	1	5
5 (most affluent)	4	0	4

Location			
Urban	18	6	24
Rural	4	0	4

**Sex**			
Male	5	0	5
Female	17	6	23

**Years since qualification**			
<5	2	0	2
5–9	9	2	11
>10	11	4	15

**Special interest in paediatrics**			
	3	3	6

IMD = Index of Multiple Deprivation quintile.^19^

### Clinical assessment and diagnostic process

HCPs reported that perceived clinical need was the most common reason for prescribing antibiotics. They determined clinical need using two diagnostic stages: first a rapid initial assessment based on pattern recognition at first sight of the child and then a more formal deductive assessment process.

HCPs described a rapid initial pattern recognition process that enabled them to recognise *‘the sick child in among the just unwell’* (Nurse Prescriber #321) as they walk through the door. The rapid initial assessment was described in terms of noting the child’s energy levels and interaction with their environment, sometimes in combination with certain symptoms (skin pallor or breathing difficulties) and sometimes described as an intuitive decision based on previous experience that could trigger, or discount, serious illness:

‘*The first thing I’m going to be looking at is what they look like as they come through the door. A lot of my decision making is based on what the child looks like.*’

(GP #326)

‘*A child who walks in upright, bright eyed, perky, starts exploring the room, even a child who walks in holding mum’s hand and sits quietly with them, is probably OK. They’ve got the energy to walk, they’ve got the energy to explore, so it doesn’t really matter what my physical findings are, they’ve still got the energy. So that in actual fact I barely need to ask mum about eating, drinking, peeing, pooing, because by and large that will be evident by looking at them. I’m much more worried about the baby who is kind of in mum’s arms, lying back, just waiting to get better.*’

(Nurse Prescriber #312)

HCPs reported not relying on the initial assessment alone, but the need for a more formal deductive assessment, including history taking and physical examination, to refine their diagnosis, and rule out serious illness. HCPs also described how their initial rapid assessment influenced the consultation. For children seen to be ‘sick’, the history taking and physical examination was vital for the HCP to form a diagnosis. For children viewed as not really ‘sick’, the physical examination was used to ensure that nothing was missed and then to focus on discovering and addressing parents’ concerns:

‘Both the parent’s report and the examination, I probably put equal weight on. The weight probably varies almost in how severe the problem is, which sounds slightly tautological. But um if the child looks well when the parent comes in, and the parent describes a cold, then the examination is to kind of rule out something more significant. If the child appears unwell then the examination becomes almost a more important part of the consultation because you’re then trying to check whether the child has a potentially serious clinical problem that may require, you know, further referral or whatever. So perhaps the balance is different between what we call the history, the patient’s or parent’s story, and the examination. And I hadn’t thought of this before, but depending on that initial assessment when they first come in.’

(GP #301)

How well the child appeared in the rapid first assessment, and whether or not certain clinical symptoms and signs were identified during the more deductive assessment process, influenced antibiotic prescribing. Opinions varied as to which clinical symptoms and signs were used to identify serious cases and prompt prescribing. The majority cited abnormal chest signs, high and persistent fever, long duration of illness usually combined with trajectory (getting worse or failing to get better), and how ill the child appeared in the initial assessment. Green phlegm, rapid pulse or breathing, vomiting, absence of URTI symptoms, and not responding to over-the-counter medication were cited by a minority:

‘If it’s been a couple of weeks already, and they think they’re no better at all and just going on the same, then I might give a prescription then … even if there weren’t really overt clinical signs.’

(GP #325)

‘Probably if I gave them [antibiotics] out would be on um children that had a fever, maybe 38.5, miserable despite having regular Calpol^®^ and Nurofen^®^, obviously on chest signs, if they were coughing up any muck, and it had to be a colour, I always have in my head like a little colour flow chart in my head, the darker the colour the more likely to be bacterium than it is if it was pale green or yellow or clear.’

(Nurse prescriber #324)

### Uncertainty in diagnosis, prognosis, and prescribing

HCPs were confident in diagnosing and managing the majority of minor and severe RTIs. However, it was the children perceived to be of intermediate illness severity who provoked the most uncertainty, and for whom HCPs often chose to prescribe rather than risk a serious RTI developing. Some HCPs said there were occasions when they prescribed antibiotics but they could not say why. Some stated they may prescribe antibiotics for all those they deemed the most serious because, as it was not possible to differentiate between a viral or bacterial cause, it was thought better to prescribe than risk not prescribing for a child who might subsequently become seriously ill:

‘There are some that are in a grey area that you’re sort of like, “Oh I’m not quite too sure.” … it might be a bit of a bacterial infection, it might not be … And in that case you may try antibiotics and see if they respond … I would say that 80% of the children you see are well or it’s a mild viral infection that you can self-manage. I’d say that maybe 5% of them are really unwell, and maybe there’s sort of er, I don’t know, 10–15% kind of grey area.’

(GP #305)

*‘I think they’re* [antibiotics] *probably prescribed more than they should be, to be quite honest. I think it seems to be quite a clinical judgement, and I think my understanding is so far that there isn’t quite enough evidence really to help us on clinical signs and history to make a diagnosis.’*

(GP #309)

‘There is uncertainty. I mean is it viral, is it bacterial? You don’t know. But you give them antibiotics, because I think you have to be quite brave if you’ve got localised chest sounds and don’t give them antibiotic.’

(GP #310)

Experience was identified by HCPs as important for increasing confidence in identifying a seriously ill child and, within those at the more serious end of the illness spectrum, in more accurately differentiating between those who needed treatment and those who could be safely monitored. Some less experienced HCPs, who had always worked in general practice, said they had not seen many children with very serious RTIs (for example, pneumonia) and had less confidence in their identification. Conversely, some less experienced GPs with secondary care paediatrics experience said their background enabled them to identify seriously ill children and consequently have confidence in not treating children at the more serious end of the spectrum who they thought would recover without treatment. In one practice, where patients would normally see the same GP each time they consulted, the HCP said that knowing the child helped them identify serious illness:

‘I certainly haven’t got experience really that much with children … It affects my confidence sometimes in dealing with children with coughs and colds … there’s always at the back of your mind, “Is this child sicker than I think they are?”.’

(GP #328)

‘What else would help me? Um more confidence in knowing symptoms and how they relate to prognosis. Um probably being better at examining children’s chests. I don’t examine many chests belonging to poorly kids, and probably if I listened to more kids with pneumonia and that sort of thing, I’d be better … I can think this is abnormal and I’m not happy, but maybe I’m not so good at um saying, “This is not quite right, but it’s not too bad, and we probably don’t need to prescribe for it”.’

(GP#301)

‘I think I’m quite fortunate because I’ve done about 18 months in paediatrics, so I’ve sort of seen the other end of the spectrum when they come in with bronchiolitis, acute asthma, that kind of stuff … it’s useful, because then I see them and I’m like “Well you look like”—you know, that’s quite engraved in my mind, because I had the luxury of that fairly extensive experience.’

(GP #304)

‘The advantage of being a GP is that you know a lot of the children and what they’re like. For example, yesterday I saw a child that’s about 1, who I have been seeing quite frequently … when the child walked in, I knew that the child wasn’t very well. She’s normally a really active child, she doesn’t let you examine her, and she was just sitting there on her mum’s lap, not doing anything, withdrawn, and just obviously, you know, not herself at all. And so that’s one of the things that you, you know, you would look out for.’

(GP #315)

Most HCPs thought that better evidence regarding prognosis, especially the evidence for the factors that could be used to distinguish the children at high and low risk of subsequent illness deterioration, would be useful in supporting their prescribing decisions:

‘It would be nice to know that if X happens it’s never a problem, or if Y happens you really want to be keeping an eye on this child, that would be nice.’

(GP #327)

### Non-clinical influences on antibiotic prescribing

When describing how they reached a decision to prescribe antibiotics, HCPs usually described additional influences other than parent-reported symptoms and physical examination findings. HCPs mentioned that if parents consulted multiple times within the same illness episode, this would increase their anxiety that there was something more seriously wrong and make them more likely to prescribe, even in the absence of the symptoms and signs they would usually use to help prescribing decisions:

‘So if someone presents repeatedly to different GPs and there’s actually nothing, no change on each occasion, by the third time someone’s going to do something because they think, “Gosh, there must be something really bad here, this person has come in three times in a fortnight, they’re obviously worried, we need to do something.” If you come often enough, something will be done because people’s anxiety goes up.’

(GP #314)

A minority of HCPs said that, if they had concerns that a parent may not re-consult if their child deteriorated, even if provided with safety net advice, they would be more likely to prescribe antibiotics:

‘If I don’t trust the mum to come back because she seems not very with it, but I’m not worried enough to admit the kid, I might be more inclined to antibiotics … Just in case they don’t come back, because I can’t safety net properly with them.’

(GP #318)

A few HCPs mentioned the pressure of time or timing of consultations in relation to access to primary health care as factors that may make them more likely to prescribe antibiotics or provide a delayed prescription to reduce parental anxiety, especially on the eve of a weekend or holiday. HCPs were also concerned with preserving a good relationship with parents and with protecting themselves from medicolegal problems, and both these would influence in favour of prescribing:

‘I give them a prescription and say, “Don’t cash the prescription in unless your child deteriorates and this, this, this, and this, in which case you might consider” … I think taking that anxiety and uncertainty out of the, “Oh crikey, you know, we’re just going down to Penzance and she’s unwell again”. I think having that, “Phew, I’ve got an umbrella in case it rains”, kind of thing, is sometimes all the parents need.’

(GP #313)

‘It’s much easier to give a prescription. If you sat there and told me this, that, and the other, you know, you’re going to be happier, I’m going to—you know, you’re going to be out the room, the system’s going to run quicker, it’s much easier to prescribe … Than to not.’

(Nurse Prescriber #323)

Most HCPs described parent pressure for antibiotics as uncommon across all patient populations. They reported that parents did not necessarily expect antibiotics and those who did were usually satisfied with an explanation and reassurance:

‘… they’re [parents] quite happy as long as you’re reassured that you can’t find anything that is definitely needing antibiotics.’

(GP #303)

‘I find it better to say to the parents at some time in the consultation, “Did you come in expecting antibiotics?” … And I think that breaks the ice quite a lot. Because the vast majority will say, “Well no I didn’t, you know, I didn’t come in for that.” And I think that sort of takes the tension out of the consultation. Because I think a lot of doctors are sort of sitting there thinking, “I don’t want to prescribe antibiotics”, and they’re thinking that the parents want antibiotics. But actually most of them don’t.’

(GP #302)

When there was perceived pressure for antibiotics, which may not have been clinically warranted, HCPs employed a range of strategies including: offering additional explanation; offering a delayed prescription; saying ‘no’; and giving a delayed prescription with advice that antibiotics were not needed now:

‘I’ve had maybe one or two that have been really difficult and won’t accept what I’m saying. But again, usually with a standby script, they’re quite happy with that because they feel they’ve got what they want. And you’ve kind of not given them quite what they want. So it’s a little bit of a compromise, but without completely destroying sort of your relationship, you know, your doctor/patient relationship.’

(GP #305)

‘If I really don’t think they need it, I try my hardest not to prescribe. If I feel it’s going to end up in a huge battle, er and um then I may say, “Well I’ll give you a prescription to keep. I would strongly recommend that you don’t go and get this at the moment, because I honestly don’t think your child needs it, and it may actually make things worse rather than better. But the prescription is there, and you’ve got it should you need it”.’

(GP #313)

## DISCUSSION

### Summary

Most HCPs decided to prescribe antibiotics based on a combination of symptoms and signs that varied, but often included how ill the child appeared, abnormal chest signs, high or persistent fever, and prolonged duration of symptoms without improvement and not in response to parental expectations. HCPs reported that they used two processes in assessing a child’s illness severity: an initial, rapid pattern recognition; and subsequent, deductive reasoning. HCPs were confident in the diagnosis and management of most minor and severe RTIs. Prognostic uncertainty, particularly for intermediate-severity illnesses, often prompted HCPs to prescribe to mitigate the perceived risk of illness deterioration. The study identified a number of non-clinical influences on prescribing (for example, parental anxiety, pressure of time, and timing of consultation) and the use of delayed prescribing as a way of increasing parental confidence to manage their child’s RTI or to maintain a good relationship with the parent. No differences were found between GPs’ and nurses’ diagnostic and antibiotic prescribing decisions working across a range of patient populations.

### Strengths and limitations

The study provides a novel, in-depth exploration of the complexities of HCPs’ antibiotic prescribing practices for children, from practices serving a range of affluent to deprived neighbourhoods. Although the participant sample was drawn from a single urban and neighbouring rural area, achievement of data saturation together with the rigour of analysis improves the credibility of findings.

### Comparison with existing literature

Perceived medical need is known to be a strong predictor of prescribing,^21^ but what has not been reported before is that HCPs make the decision whether or not to prescribe antibiotics after a two-stage assessment process, which echoes Djulbegovic and colleagues’ dual processing model of medical decision making.^22^ In this model, system 1 is an initial, fast, intuitive assessment based on previous experience of the condition, which has also been categorised as ‘spot diagnosis’.^23^ System 2 is a slower, analytical, deliberative, and logical assessment. HCPs evaluate the relative benefits and risks of treatment using both system 1 and system 2 thinking, and each may moderate the other. In this current study, system 1 was used to identify obviously ‘serious’ or minor cases and then system 2 was used to double check the system 1 diagnosis and to aid decisions as to whether cases would benefit from antibiotics. Previous studies of the clinical decision-making process have focused on the symptoms and signs associated with antibiotics prescribing,^15^,^24^ which is part of the system 2 process. This current study identifies the importance of the intuitive system 1 rapid pattern recognition in clinician decisions about whether or not to prescribe antibiotics.

Although there was a general consensus among HCPs in this study around the core symptoms that normally prompted an antibiotic prescription (chest signs, fever, longer duration, worsening trajectory, and how ill the child appeared), there was also variation in practice with some prescribing for clinical symptoms and signs that others felt did not indicate antibiotics. Brookes-Howell’s^15^ multicountry qualitative study found a general consensus around the clinical signs (abnormal chest sounds, fever, coloured sputum, and breathlessness) for which HCPs prescribed in adults, although these differed slightly from those identified in the current study of children. Some HCPs reported that an acute cough lasting >2 weeks may prompt them to prescribe antibiotics. National Institute for Health and Care Excellence guidelines estimate a 3-week average duration for an acute cough,^25^ which contrasts with a recent systematic review which found that acute cough symptoms are better in 50% of children by 10 days.^26^ HCPs also prescribed antibiotics for certain clinical signs (for example, coloured sputum) even when there is not strong evidence linking them to bacterial infection.^15^,^24^ In the absence of clear clinical guidelines or up-to-date evidence that recommend treatment strategies, many HCPs have developed personalised ‘rules of thumb’, leading to between–HCP variation and confusion for patients and parents.^27^ This study described the diagnostic and prognostic complexity of RTIs, with HCPs using a range of symptoms and signs to guide them but not always being able to rule out serious illness. Many HCPs recognised that differentiating between bacterial and viral infection was not possible, and in the face of this uncertainty HCPs generally favoured prescribing. Diagnostic uncertainty has previously been associated with increased likelihood of antibiotic prescribing^3^ as a way to attempt to protect patients from subsequent illness deterioration.^9^,^27^,^28^ Over-prescribing of antibiotics for children has been justified, in part, by the risks of complications in children if they did not prescribe.^9^,^29^ Previous research has suggested that past negative experiences may make HCPs more cautious and increase prescribing behaviours.^27^ The findings here suggest that increasing primary care HCP experience of children with serious RTIs, particularly HCPs with limited exposure to paediatrics in secondary care, may increase clinical confidence and help reduce prescribing.

Previous research has focused on the influence of parental pressure on unnecessary antibiotic prescription^9^ but there is evidence that parents are primarily seeking a medical evaluation when they consult and defer the treatment decision to the clinician.^10^,^11^ It was found that HCPs reported that parent pressure was rare and outweighed by clinical uncertainty and the non-clinical factors influencing prescribing. Many of the non-clinical influences that were found are similar to those described previously, including HCPs prescribing because it is quick and easy without endangering the relationship with patient/parent.^28^,^30^–^33^ As with this study, previous research describes how an anxious patient or concerned family member can transmit the anxiety to the HCP,^33^,^34^ who then may be more likely to stray from clinical guidelines.^33^ What this study adds is the influence of low clinician confidence in the ability of some parents to spot a deteriorating child on the HCP’s decision to prescribe antibiotics. Further improving parental safety-netting advice could reduce the need to prescribe ‘just in case’. More evidence regarding the optimal format for delivering safety-netting advice is needed.^35^ However, the use of a multifaceted and tailored approach to safety netting is encouraged,^36^ with parental information leaflets being a promising tool for reducing antibiotic prescriptions.^37^

### Implications for research and practice

Despite the publication of clinical guidelines,^25^,^38^ this study suggests more evidence is needed to support clinical decision making and reduce diagnostic uncertainty and variation in antibiotic prescribing for childhood RTIs. HCPs require more detailed evidence about the prognosis of RTIs in children, especially those of intermediate severity, in order to support HCPs to identify those children most and least likely to benefit from antibiotics. Some HCPs may benefit from additional training in paediatrics, particularly those with limited experience of children with serious RTIs, to increase confidence in non-antibiotic treatment strategies rather than prescribing in the face of uncertainty.
